# The acceptability to patients of PhysioDirect telephone assessment and advice services; a qualitative interview study

**DOI:** 10.1186/s12913-016-1349-y

**Published:** 2016-03-28

**Authors:** Jennifer Pearson, Jane Richardson, Michael Calnan, Chris Salisbury, Nadine E. Foster

**Affiliations:** Faculty of Health & Applied Sciences, University of the West of England, Glenside Campus, Blackberry Hill, Bristol, BS16 1DD UK; Arthritis Research UK Primary Care Centre Research Institute for Primary Care and Health Sciences, Keele University, Keele, Staffs ST5 5BG UK; School of Social Policy, Sociology and Social Research, University of Kent, Cornwallis North East, Canterbury, Kent CT2 7NF UK; NIHR School for Primary Care Research, School of Social and Community Medicine, University of Bristol Canynge Hall, 39 Whatley Road, Bristol, BS8 2PS UK

**Keywords:** Physiotherapy, Service delivery, Patient experience, Interview, Qualitative study

## Abstract

**Background:**

In response to long waiting lists and problems with access to primary care physiotherapy, several Primary Care Trusts (PCTs) (now Clinical Commissioning Groups CCGs) developed physiotherapy-led telephone assessment and treatment services. The Medical Research Council (MRC) funded PhysioDirect trial was a randomised control trial (RCT) in four PCTs, with a total of 2252 patients that compared this approach with usual physiotherapy care. This nested qualitative study aimed to explore the acceptability of the PhysioDirect telephone assessment and advice service to patients with musculoskeletal conditions.

**Methods:**

We conducted 57 semi-structured interviews with adults from 4 PCTs who were referred from general practice to physiotherapy with musculoskeletal conditions and were participating in the PhysioDirect trial. The Framework method was used to analyse the qualitative data.

**Results:**

The PhysioDirect service was largely viewed as acceptable although some saw it as a first step to subsequent face-to-face physiotherapy. Most participants found accessing the PhysioDirect service straightforward and smooth, and they valued the faster access to physiotherapy advice offered by the telephone service. Participants generally viewed both the PhysioDirect service and the physiotherapists providing the service as helpful. Participants’ preferences and priorities for treatment defined the acceptable features of PhysioDirect but the acceptable features were traded off against less acceptable features. Some participants felt that the PhysioDirect service was impersonal and impaired the development of a good relationship with their physiotherapist, which made the service feel remote and less valuable.

**Conclusion:**

The PhysioDirect service was broadly acceptable to participants since it provided faster access to physiotherapy advice for their musculoskeletal conditions. Participants felt that it is best placed as one method of accessing physiotherapy services, in addition to, rather than as a replacement for, more traditional face-to-face physiotherapy assessment and treatment.

## Background

Musculoskeletal pain problems are extremely common in the population. Up to 30 % of all general practitioners’ (GPs) consultations in the UK involve a musculoskeletal problem, and over a quarter of registered patients will consult their GP for a musculoskeletal problem in a one-year period [1]. Many people with musculoskeletal problems are referred to physiotherapy, with approximately 6.7 million new referrals made to physiotherapy services each year in the National Health Service (NHS) [2, 3]. However, arrangements concerning how and when people with musculoskeletal pain access physiotherapy services vary across the UK, depending on local circumstances. This often means physiotherapy services have long waiting lists, resulting in waits for treatment from several weeks to months. Several initiatives have been developed to help address this problem, including the introduction of physiotherapy-led telephone assessment and advice services known as ‘PhysioDirect’. These services allow patient to contact a physiotherapist who will then assess their musculoskeletal symptoms over the telephone.

The PhysioDirect service tested in RCT is a typical service model. The participants involved in the RCT were invited to telephone a senior physiotherapist. Participants were either provided advice about good self-management over the telephone, and posted a relevant advice leaflet and appropriate exercises or they were invited to attend a face-to-face appointment at their local physiotherapy department. Participants who solely managed over the telephone were encouraged to phone back to report progress after two to four weeks, and if they re-contacted the service they were re-assessed and given further advice or a face-to-face consultation was arranged if it was felt necessary [4]. Thus, PhysioDirect was a service that provided a package of care, rather than only telephone assessment and advice.

The results from a randomised trial of PhysioDirect showed that it was safe, resulted in equivalent clinical outcomes (participants’ physical function) [4]. The trial also found that participants who were randomised to PhysioDirect were no more satisfied with access to physiotherapy than usual care participants, but had slightly lower satisfaction with regards to the consultation and overall satisfaction at six months. However, a limitation of satisfaction surveys is that they do not describe the patient experience [5]. From the patient perspective, healthcare acceptability is linked to how patients experience and evaluate the quality of care they receive [6]. In relation to telehealth, acceptability is evaluated in terms of patients’ physical and psychological comfort with the application, the convenience of the encounter, the personal skills and the manner of the professional, the assessment of the lack of face-to-face contact and the willingness to use service again [7]. This qualitative study investigated how patients experienced the PhysioDirect service, with the main aim of exploring its acceptability from their point of view. The main objective of this paper is to describe the key variables that determined patient acceptability of the PhysioDirect service and to understand how the patient experience differed from those accessing usual physiotherapy care. The perceptions of physiotherapists, managers, GPs and commissioners are reported elsewhere [8].

## Method

The qualitative study was nested within PhysioDirect RCT and details of the methods, clinical and economic results of the trial are available elsewhere [4, 9, 10]. Full ethical approval was granted for the study from Southmead Research Ethics Committee, Reference 08/H0102/95 and full PCT Research and Development (R & D) approval was granted by each PCT prior to the start of the RCT. All the participants in the qualitative study provided written and informed consent prior to being interviewed.

### Sampling

The criteria for the selection of a purposive sample of interview participants from the wider sample participating in the RCT were as follows: PCT, trial arm, gender, age and site of musculoskeletal complaint (see Table 1: characteristics of sample). Participants were sampled in order to include individuals from four different groups in the qualitative interviews; those randomised to the PhysioDirect service who proceeded to have telephone contact only, those randomised to the PhysioDirect service who had both telephone contact and then face-to-face contact by a physiotherapist, those randomised to PhysioDirect but who subsequently chose not to telephone the service and finally, those randomised to usual physiotherapy care. It was particularly important to understand the acceptability of the PhysioDirect service from those patients who received the telephone contact only and therefore, more of those patients were selected for interview. Usual care patients were interviewed in order to facilitate comparisons with the new PhysioDirect service. Those who randomised to PhysioDirect but chose not to contact the service provided useful insights as to whether the service was unacceptable. Participants were sampled to ensure a breadth of age, gender, PCT, site of musculoskeletal complaint across the four patient groups.

**Table 1:** Summary of participant characteristics according to the interview sampling criteria

Participant characteristics		Number	Percentage %
Gender	Male	26	46
	Female	31	54
Age	Mean (SD)	58 (16.88)	
	Range	19–87	
Trial arm and patient group	PhysioDirect arm: telephone care only	25	44
	PhysioDirect arm: telephone and face-to-face care	13	23
	PhysioDirect arm: did not contact the service	10	17
	Usual physiotherapy care arm	9	16
PCT	PCT A	17	30
	PCT B	15	26
	PCT C	13	23
	PCT D	12	21
Site of musculoskeletal complaint	Lower limb	23	40
	Upper limb	14	21
	Cervical spine	5	25
	Lumbar spine	12	9
	Multiple areas of pain	3	5

### Participants and interview process

The main PhysioDirect trial database was used to identify potentially eligible participants for the nested qualitative interviews. Participants meeting the relevant criteria were identified from the database and invited by letter to take part in the qualitative interviews. In total, 388 participants were invited to take part in the qualitative interviews over a period of 9 months from August 2009 to April 2010. 82 agreed to be interviewed, however, thematic saturation was reached after 57 interviews. Participants were selected based on our sampling criteria and their availability to attend the interview. Each participant was interviewed once, soon after their physiotherapy episode of care in the RCT, at a convenient time and location to the participant (mostly participants’ homes).

The interviews were semi-structured, guided by a literature informed topic guide developed by the study team (see Appendix). The topic guides were reviewed following early interviews so that new questions were incorporated in response to unanticipated themes arising from earlier interviews [11]. This process of topic guide amendment was followed for each of the four patient groups interviewed. In addition to the topic guide reflection and amendment, interviews were listened to, transcripts were re-read to check for accuracy and to glean initial ideas about issues of potential importance.

### Data analysis

A Framework approach was used to analyse the qualitative data [12]. The Framework approach is a thematic, cross-sectional analysis that allows the researcher to simultaneously analyse across themes and cases. It enables qualitative findings and interpretations to build from the original data, allowing the analysis to maintain a clear auditable trail. The approach is a matrix-based method for analysing qualitative data that includes familiarisation with the data, the creation of a theoretical framework, indexing the data according to the Framework and the creation of summaries from the indexed data. These summaries are then finally mapped in charts and descriptive explanatory accounts are created. The audio files of the patients were listened to several times and transcripts reread to identify key themes and concepts. The data were sorted and reduced to a manageable form, and a theoretical index was refined to summarise the essence of the transcripts. In order to ensure confirmability and trustworthiness, a sample of transcripts was double coded and the thematic framework was reviewed by four of the authors (JP, JR, MC and NF) before it was applied to all data. A data summary was attached to each of the data labels on the index. Large charts of the index headings and attached summaries were created and descriptions that captured the essence of the summarised data across the cases were made with key themes identified. Finally, the themes were mapped and interpreted by the author team in order to construct overall explanations of the data.

## Results

In total, 57 face-to-face semi-structured interviews were conducted, each lasting on average 43 min (ranging from 14 min and 07 s to 66 min and 38 s). Table 1 provides a summary of the participants who took part in interviews, showing their key characteristics according to each of the sampling criteria. Slightly more women were interviewed than men, and although the average age was 58 years, there was a large range of ages, with the youngest person interviewed being 19 years and the oldest being 87 years. More participants were interviewed in the group that was randomised to the new PhysioDirect service than in the usual-care group, including those who received some or all of the components of the new service, as well as those who were randomised to the new service but who never telephoned or contacted the service. Nine interviews were conducted with patients randomised to usual care. A range of participants with similar characteristics were interviewed across the four patient groups.

In addressing the acceptability of the PhysioDirect service, we grouped the 13 sub-themes into 4 overarching themes: expectation of the PhysioDirect service, PhysioDirect as an ‘access point’ into physiotherapy, acceptable features of the PhysioDirect service and less acceptable features of PhysioDirect. Details are summarised in Table 2 along with descriptions of the themes and illustrative quotes from participants. All participants names used are pseudonyms.

**Table 2:** Summary of key themes, descriptions and illustrative quotes describing the acceptability of the PhysioDirect service

Theme	Description	Illustrative quote
Expectations of the PhysioDirect service		
Physiotherapy is a physical intervention	The belief that physiotherapy is “hands on” and therefore could not be accomplished over the telephone.	*“Well, you wouldn’t call it physiotherapy would you, not over the phone. You can’t do physiotherapy over the phone, can you?”*
		Steve, age 40, PhysioDirect arm: telephone care only
PhysioDirect can deliver physiotherapy	Initial scepticism of the PhysioDirect a belief that the service can provide physiotherapy.	*“I think, on second thoughts, you know, after I’d done it that wasn’t really so necessary, that whoever you were talking to would be expert enough to understand how the pain affected you and whereabouts and as you described it. This is a condition they must meet with over and over again I would have thought. That was just an initial response. I think on reflection it’s quite good actually.”*
		Giro, age 80, PhysioDirect arm: telephone care only
No expectations	Patients had no expectations of the PhysioDirect service.	*“No, I didn’t. I had no expectations whatsoever. I didn’t know what it would be like.”*
		James, age 63, PhysioDirect arm: telephone care only
PhysioDirect as an ‘access point’ to physiotherapy		
Direct access	Patients got through to the service without any difficulty.	*“I got through alright, there was no problem getting through.”*
		Walter, age 79, PhysioDirect arm: telephone care only
		*“I must have picked a convenient time because she just answered the phone.”*
		Lynn, age 69, PhysioDirect arm: telephone care only
Call-back service	Patients rang the service and were offered a call-back at a time that was acceptable to them.	*“I phoned this number, she took my details, telephone number and said I will get the person to phone you back and that happened within the hour.”*
		Somerton, age 51, PhysioDirect arm: telephone care only
		*“Yeah, I got through without problems. She was busy at the time and, just asked could I, would it be alright if they phoned back later in the afternoon.”*
		Peter, age 74, PhysioDirect arm: telephone care only
Difficulty in access	Problems arose when the PhysioDirect service was busy and patients were unable to get through.	*“It took quite a bit to get through. That was a bit annoying. It took several calls to get through.”*
		Lucy, age 53, PhysioDirect arm: telephone care and face-to-face contact*.*
		*“There was a little bit of a problem, to get through.”*
		Wendy, age 58, PhysioDirect arm: telephone care only
No access	Explanations as to why patients did not contact the PhysioDirect service.	*“It was, yeah, it was basically because I was going on holiday that, they were going to do it on the phone but I was going on holiday on the Friday or the Saturday and it was getting worse, my back and I thought I’ve got to do something, I’ve got to drive down to PLACE, you know and that’s why I went private.”*
		Brian, age 48, PhysioDirect arm: did not contact the service
		*“The only reason I didn't phone was because, you know, the injury was gone and I just didn't feel like I needed to, sort of, take that step forward.”*
		Harry, age 23, PhysioDirect arm: did not contact the service
		*“I haven’t been able to get in touch because it doesn’t fit into the criteria of a person who is working.”*
		Pauline, age 43, PhysioDirect arm: did not contact the service
		*“Because I think that arthritis can probably be treated in a better way. I do exercise quite a lot, I do walk and do that sort of thing. I don’t think physiotherapy would be getting to the root problem.”*
		Hannah, age 65, PhysioDirect arm: did not contact the service
		*“It comes to a time when you think bugger it, I can’t be bothered, you know. It’s just too much, for me, it’s just too difficult to try and get out of here, get to the doctors to try and find out, get an appointment with them, come back.”*
		Aarron, age 42, PhysioDirect arm: did not contact the service
Acceptable features of PhysioDirect		
Quick and convenient service	The PhysioDirect service was perceived as quick, efficient and reduced the time to speak to a professional about their problem.	*“Well, the thing I liked about it really, it didn’t take long for them to get in touch with me.”*
		Mary, age 76, PhysioDirect arm: telephone care only
		*“The immediacy of it was good.”*
		Helen, age 59, PhysioDirect arm: telephone care only
		*“It was quick. That was the, um, it seemed to plug the gap of having to wait for an appointment.”*
		Peter, age 74, PhysioDirect arm: telephone care only
The helpful physiotherapist	The PhysioDirect physiotherapists were perceived as being a positive, helpful, polite, pleasant and knowledgeable.	*“I found her very clear, thorough and very pleasant. She was very pleasant. She really was good.”*
		Wendy, age 58, PhysioDirect arm: telephone care only
		*“Very helpful, very nice. Yes. Very helpful.”*
		Lynn, age 69, PhysioDirect arm: telephone care only
		*“She was very good.* S*he asked me a lot of questions to enable her to be able to get a good diagnosis over the phone.”*
		Peter, age 74, PhysioDirect arm: telephone care only
PhysioDirect was effective at providing self-management advice	The PhysioDirect service provided advice and information enabling participants to self-manage their musculoskeletal condition.	*“It’s a good thing because obviously, not everybody knows the best way in order to aid their injury. When I hurt my ankle and they sent out the information to me after the initial over the phone consultation with the PhysioDirect, they sent me out a book of all the different exercises in order to aid my ankle.”*
		Robert, 30, PhysioDirect arm: telephone care only
Less acceptable features of the PhysioDirect service		
PhysioDirect was an ‘impersonal’ service	The PhysioDirect service was perceived as an impersonal service.	*“Because it’s a face-to-face, personal thing. You know that there’s somebody sitting there waiting for you turn up and you don’t or you’re cancelling your appointment that somebody’s gone to the trouble to make for you, whereas a phone call’s just a phone call and it can be anytime and anywhere, so, it’s less personal.”*
		Hannah, age 65, PhysioDirect arm: telephone care only
		*“Well, you know, somebody out of the ether is talking to you, not like you laughing like that or something like that, it is simply not personal enough. It’s simply not personal enough. And, I know they’ve got a lot of work to do but that doesn’t make me feel any better.”*
		William, age 81, PhysioDirect arm: telephone care only
		*“I just mean somebody who, you know, I just feel that this PhysioDirect, you are just a number on a piece of paper, but, like I say, if you rang me back in a month’s time and actually had a conversation with me, I would feel that that was more personal.”*
		Faith, age 52, PhysioDirect arm: telephone care only
Communication difficulties	The PhysioDirect service impaired effective communication between the participant and the physiotherapist.	*“Yeah, I found it a bit, quite difficult, because it’s hard to explain isn’t it, even, not just on the phone but to anybody. I mean, the pain I was in was really, really bad, so, um, I would have preferred to have saw somebody, you know, because when you try and explain the areas or, you know, where the pain was, which it goes all the way down, down to there, it’s a bit hard to describe on the phone, so, that’s when I would have liked to have seen somebody.”*
		Jenny, age 36, PhysioDirect arm: telephone care only
Trade-offs	Participants made trade-offs between the accepted and less accepted features of the service.	*“Not having somebody there seeing how far you can bend it or move it in a certain direction just takes a little bit of the personal side out of it. But, you know, on the flip side, it takes a lot of the time waiting to be able to see a physiotherapist.”*
		Robert, age 30, PhysioDirect arm: telephone care only
		*“I mean, you have to make the journey, you have to go, you have to sit there, you very rarely get in at the time of your appointment, you usually wait half an hour, more, um, then you go in and you're in strange surroundings whereas on the telephone, you're in your own home, it's immediate, you have no waiting time.”*
		Lynn, age 69, PhysioDirect arm: telephone care only

### Expectations of the PhysioDirect service

Participants’ expectations of PhysioDirect influenced whether they engaged with the service and how they evaluated it. Some participants had firm ideas of what they expected from physiotherapy, perceiving it to be a physical treatment that is done to them and therefore, these participants felt that the PhysioDirect service could not meet their needs.

*“I thought I might get some advice on the phone which means I can start early before my appointment and I was actually quite surprised I didn’t get an appointment at all.”* Lucas, age 34, PhysioDirect arm: telephone care only

However, there were participants who were initially sceptical of PhysioDirect, but who changed their minds and viewed it positively after they actually experienced the service. It appears that their opinion had changed from the perception that ‘proper’ physiotherapy was impossible via the telephone, towards understanding that effective physiotherapy assessment and advice could be telephone based. Others who were interviewed felt they had no prior expectations of the PhysioDirect service. Many of these participants had no previous experience of physiotherapy and may therefore have been less likely to expect face-to-face contact.

### PhysioDirect as an ‘access point’ into physiotherapy

The qualitative interviews with participants reflected the range of possible experiences when accessing the PhysioDirect service and were categorised into four distinct groups: direct access, call-back service, difficulty in access and failed to access. Of those interviewed who had been randomised to the PhysioDirect arm in the trial (*n* = 38), 25 experienced telephone contact only and 13 both the telephone contact and face-to-face care. From those 38 participants, 13 spoke to a physiotherapist who assessed them immediately, 12 participants experienced the PhysioDirect service as a call-back service, 5 participants described difficulties in accessing the service, but after persisting in calling they were eventually successful.

Participants perceived PhysioDirect as an early stage in the process of accessing physiotherapy and referred to the PhysioDirect service as the first stage in accessing physiotherapy treatment. They described the telephone consultation with the physiotherapist as the first step in this process. Participants also perceived that the PhysioDirect service already existed within the healthcare system and that the level and mode of input from physiotherapists would increase, depending upon the complexity of the problem. The second stage of care was described by participants who were invited for a face-to-face appointment.

*“You’ve got to try something to see if you can resolve the problem and it’s easier to resolve it in the simplest ways rather than go into the extreme ways, because maybe you don’t need to go to the extreme, you can do the first stage first and that maybe resolves it. Or maybe you might have to go to the second stage and that resolves it.”* Somerton, age 53, PhysioDirect arm: telephone care only

Some participants accepted that the PhysioDirect service provided the ‘first stage’ of physiotherapy care, whilst others felt that the PhysioDirect service introduced an unnecessary stage which actually impaired their access to ‘proper’ physiotherapy services.

*“It’s just annoying. Well, I’ve done that and as far as I’m concerned now I’ll ring up tomorrow and say ‘What’s the next stage, I’m not happy with what’s happening, are you proposing anything else or do I have to go back to the doctor and see what he can do?’ Because, as far as it is at the moment, it’s a waste of time. It’s done nothing for me at all.”* Walter, age 79, PhysioDirect arm: telephone care only

The quantitative trial data showed that 85 % of patients in the PhysioDirect arm contacted the service at least once [4]. Therefore, 15 % of patients who were randomised to the PhysioDirect service and consented to take part in the trial did not contact the PhysioDirect service. It was important for the qualitative interview study to try to understand why these patients chose not to contact the service. The reasons provided by participants included difficulty accessing the service due to inconvenient opening hours (during working hours for participants); the perceived cost of the telephone call for some participants; some chose to seek out private physiotherapy instead; the musculoskeletal problem had resolved; low expectations about the benefit of physiotherapy; and for some participants, other competing priorities in their lives meant that they did not prioritise contacting the PhysioDirect telephone service. Of course, failure to take up the offer of physiotherapy is not a unique feature of the PhysioDirect service. In our sample of patients randomised to usual physiotherapy care who took part in the interviews (*n* = 9), three did not attend their physiotherapy appointment despite being a participant in the trial. The first participant did not attend the face-to-face physiotherapy appointment due to the length of the wait from GP referral to appointment and they sought out private physiotherapy instead. The second participant had moved address and his address details were lost to the physiotherapy service therefore, he was unaware of his usual care appointment date. The third participant failed to attend her appointment but was already in contact with the physiotherapy department due to a previous injury for which she had received treatment. She reported that she intended to contact the physiotherapy department that treated her previously in the near future.

### Acceptable features of the PhysioDirect service

The most acceptable features of the PhysioDirect service expressed by participants were that it was quick, efficient and convenient. Participants preferred the immediacy of the telephone advice compared to the longer waiting times for face-to-face physiotherapy care (in the trial patients in the PhysioDirect arm had their first telephone contact on average 7 days after randomisation, versus 34 days for face-to-face care in the usual care arm) [4]. They felt that NHS physiotherapy waiting lists were too long and suggested that a wait of two weeks from the date of the GP referral to the first physiotherapy contact would be more acceptable.

“*It’s not a viable proposition to say I’m gonna go to the physio tomorrow. Um because life isn’t like that but certainly I would have thought within one or two weeks um of being referred and you should have had some form of consultation done within that period of time, you know to even to turn around say well all you need is exercise you know or whatever.”* Kurt, age 61, PhysioDirect arm: usual care

Participants also liked being able to access the PhysioDirect service in their own homes and places of work, and described not having to go to the physiotherapy clinic, take time off work or pay for car parking as convenient. The telephone style of the physiotherapists providing the PhysioDirect service was also perceived by participants to be very important. The participants interviewed were very positive about the physiotherapists in both the usual care and PhysioDirect arms. Participants perceived the physiotherapists to be polite, helpful and friendly. They described their physiotherapist as the knowledge provider, able to advise, provide information on their pain condition and offer time frames for the participant to phone back if their problem did not improve as expected. The PhysioDirect service was perceived by participants as effective in providing self-management advice and was described by participants as providing them with the knowledge to carry out their own physiotherapy at home.

*“It was the fact knowing that that person, sort of, seemed to understand what you were going through and just trying to be helpful and give you advice and then it’s left for you to try it and then take it from there and then if there’s a problem that person would still be there to phone and get more advice on it if you needed it.”* Somerton, age 51, PhysioDirect arm: telephone care only

### Less acceptable features of the PhysioDirect service

The most common negative feature of PhysioDirect perceived by participants was that the telephone care was felt to be impersonal. The words ‘not personal’ were used by participants in their narrative when they described the features they disliked about PhysioDirect. For some participants the one-off assessment and treatment advice in the PhysioDirect service, appeared to contribute to their perceptions that PhysioDirect was impersonal as there was no ongoing contact with the therapist. There was no evidence in the interviews with usual care participants that they viewed the usual face-to-face physiotherapy service as impersonal. A further concern of those interviewed was the absence of non-verbal communication in the PhysioDirect service, i.e., the lack of visual cues and physical contact. The lack of visual cues made it difficult for participants to explain to the therapist where exactly they were experiencing their pain. For example, in a face-to-face consultation, the participant could explain the location of pain by physically pointing to the painful spot on their body, pinpointing the exact anatomical position. Most participants found it difficult to explain the bodily movements they were making in order to feed back to the physiotherapist over the telephone during the assessment of their musculoskeletal condition. Reliance upon their own descriptions and interpreting what the physiotherapist said and meant resulted in the assessment creating some doubt in the minds of the participants about whether they had described their problem sufficiently well for the physiotherapist to make an accurate diagnosis. Some participants reported that they had unanswered questions about the advice and information they received over the telephone, and whilst participants could telephone the service again to clarify, some seemed reluctant to do that.

*“It left me with more questions and like I said, although I knew I could phone them, I didn't want to talk to someone on the phone. I wanted to be able to sit opposite someone face-to-face and say X, Y, Z, you know.”* Helen, age 59, PhysioDirect arm: telephone care only

None of the usual care patients interviewed reported finding it difficult to describe their symptoms to their physiotherapist during their face-to-face assessment. It also appeared that many of the participants who expressed difficulty with explaining their condition over the telephone were invited by the physiotherapist for a face-to-face appointment.

The interview data also highlighted that participants made trade-offs between the acceptable and less acceptable features of the PhysioDirect service, evaluating and weighing-up the different aspects of their experience. The most acceptable feature of PhysioDirect was the speed of access to physiotherapy assessment and advice. Participants’ trade-offs centred upon the perception that the PhysioDirect service resulted in faster access to physiotherapy than waiting for a face-to-face appointment, and this was viewed as a sufficient benefit to accept the reduction in personal (face-to-face) contact.

## Discussion

The evidence from the qualitative interviews showed that participants experienced the PhysioDirect service as quick and efficient. These findings should be interpreted alongside the findings from the randomised trial that patients in the PhysioDirect arm received their initial assessment by a physiotherapist more quickly than those in the usual care arm, yet did not express greater satisfaction with access to physiotherapy [4]. Although participants valued fast access to physiotherapy advice they also perceived the PhysioDirect service as the first stage in accessing physiotherapy with talking on the telephone to a physiotherapist as the first step in this process and the face-to-face consultation as the second stage. This suggests that the PhysioDirect service provides a useful option or choice for people wanting early advice from a physiotherapist rather than as a replacement for face-to-face care. Similar findings were concluded by Pinnock and colleagues who suggested that the perceived benefits of telephone based care compared to face-to-face consultation resulted in a recommendation that asthmatic patients in general practice should be offered a choice of consultation [13]. More broadly, patient acceptability of NHS Direct and telephone triage in primary care have been demonstrated [14, 15]. In addition, telephone based services providing healthcare support for the management of long-term conditions such as diabetes are also acceptable [16].

Some participants described the PhysioDirect service as ‘remote’ and ‘impersonal’ and some found it difficult to describe their symptoms adequately over the telephone. It is acknowledged that describing pain is often challenging [17]. However, it appears that describing symptoms over the telephone, rather than being able to also physically show the physiotherapist the impact of their symptoms, exacerbated the difficulty that participants had in describing their pain. Participants also reported that they sometimes felt that the telephone consultation impeded the therapeutic relationship between the participant and physiotherapist. Inter-personal care and communication between patients and health care professionals are important in how patients judge quality [6], and have also found to be one of the most powerful influences on levels of patient satisfaction [18, 19]. This might explain why the PhysioDirect telephone consultations often were perceived to be of less value than a face-to-face consultation. Importantly the data showed that in order for the PhysioDirect service to be acceptable, participants needed to make trade-offs between speed of access to physiotherapy advice and reduction in personal contact. Patients make similar trade-offs when patients decide to consult their GP in primary care [20–22]. Whilst trade-offs are an important concept to highlight, if waiting times were reduced to a maximum of two weeks for face-to-face physiotherapy, the trade-offs made by participants for speed of access may mean that the PhysioDirect service would perhaps no longer be considered acceptable.

The data showed some participants who were initially sceptical and have negative expectations of the PhysioDirect service changed their mind after they experienced it, evaluating it as positive. It was likely that the acceptable features of the PhysioDirect service, for example, the physiotherapists, improved access and treatment outcome influenced their subsequent evaluation of the service. Nevertheless, the qualitative results highlight that for participants who expected to be seen by a physiotherapist were more likely to evaluate the PhysioDirect service as unacceptable. It might be safe to assume that those participants who had strong expectations of being seen face-to-face also had preferences, before the start of the trial, to receive usual physiotherapy care. However, the trial tested for an interaction between baseline participant preferences and randomisation arm in terms of satisfaction with the service and found no significant interactions [4]. The qualitative results, nevertheless, showed that if participants expected face-to-face care and did not receive it they were dissatisfied and tended to evaluate PhysioDirect as unacceptable. Patients’ expectations are thought to moderate the relationship between patient concern and satisfaction [23]. Therefore, for successful implementation of the PhysioDirect service elsewhere, it seems particularly important for physiotherapists to clearly communicate to patients the role and function of the PhysioDirect service, to increase its acceptability and to elicit strong preferences for face-to-face contact where they exist.

### Strengths and limitations

The strength of this study was that the patient sample reflected the wide range of participants who used physiotherapy services across the four PCTs involved in the trial. This provided the qualitative study with a rich dataset to explore the acceptability of the PhysioDirect service. An additional strength of this study was that non-users views were sought to fully explore the acceptability of the new PhysioDirect service. The views of usual care patients established usual physiotherapy care in participating PCTs which allowed a direct comparison to those patients who experienced the PhysioDirect service. Additionally, views were also collected from patients who were randomised to PhysioDirect trial arm but did not contact the service as it was important to establish if there were any practical or theoretical reasons as to why the PhysioDirect service might be unacceptable. All the participant interviews were carried out face-to-face.

A limitation was the proportion of participants agreeing to be interviewed from the total number invited. It was felt that in order to fully explore the acceptability of the PhysioDirect service it was important to include a wide range of participants with different characteristics, in the interviews. It was difficult in some cases to arrange interviews at mutually convenient times, in addition some participants cancelled their previously arranged interviews. Another further limitation is that only three participants were interviewed who were randomised to usual physiotherapy care but failed to attend their physiotherapy appointment. This made it difficult to make any conclusions about the comparison with those participants randomised to the PhysioDirect service who did not telephone or contact the service. Thus it is unknown whether there are similar or different explanations for patients not accessing the PhysioDirect service in comparison to those not accessing traditional face-to-face physiotherapy.

## Conclusion

Many participants felt that PhysioDirect was a useful option for those wanting early physiotherapy advice for their musculoskeletal condition. However, they tended to view PhysioDirect as a useful first stage in the assessment and advice process rather than as a replacement for face-to-face care. Participants’ expectations of PhysioDirect influenced how they evaluated the service, and these expectations were often based upon their previous experience of physiotherapy and on their views of what constitutes good physiotherapy. The acceptability of PhysioDirect was in part determined by the manner in which participants traded off fast access to physiotherapy advice on the telephone versus the lack of personal contact. These findings support the quantitative trial results [4] that PhysioDirect is a useful option for services to consider but it is unlikely to merit becoming the only mode of access to physiotherapy in the future.

## Appendix

### Topic Guide PD Call only: Version 1

**Aim and objectives:**

The overall aim of this study is to explore the acceptability of a new way of delivering physiotherapy services. Experiences of and views about how patients’ experience physiotherapy are of particular interest.

**Background information:**

For the context of the interview it would be helpful to know some brief information about you. Can you give me some background information about yourself? For example, what you do for a living? What do you enjoy doing in your spare time?

**Problem:**

Can you tell me about the problem you were referred to physiotherapy for? For example, how long have you had the problem? How has it affected you in your day-to-day life?

**Process to physiotherapy:**

Can you tell me how you were referred to physiotherapy?

**Physiotherapy expectations:**

I would like to know if you have ever had physiotherapy before? Can you tell me about your experience?

**Physiotherapy attitudes, beliefs:**

I would like to know what you think about physiotherapy? Did you think physiotherapy would help your problem?

**Point of contact:**

Can you tell me how you contacted the service?

For example, opening times, ease of access

**Overall experience:**

I would like to know about your experience of talking to someone over the telephone.

For example, consultation, physiotherapist, information and advice, call length

**Outcomes:**

I would like to know if the physiotherapy you received has helped your problem?

**View of the service:**

I would like to know what you thought of the service and what you liked and disliked about it? Is there anything you would change about the service you received? Would you use the PhysioDirect service again? What impact has the service had the service had on your problem if any?

**Future suggestions:**

I would also like to get your views on accessing other services via the telephone. Do you telephone bank? Have you ever used NHS Direct or the GP out of hour (OOH) services?

**Closing:**

Thanking re information given, reflection on what was said, and other questions?

**Consent:**

Reiterate confidentiality and thank

## Abbreviations

CCG: Clinical Commissioning Group
GP: General Practitioner
MRC: Medical Research Council
NHS: National Health Service
PCT: Primary Care Trust
R&D: Research and Development
RCT: Randomised Control Trial
